# Diverse Task Classification from Activation Patterns of Functional Neuro-Images Using Feature Fusion Module

**DOI:** 10.3390/s23073382

**Published:** 2023-03-23

**Authors:** Osman Tayfun Bişkin, Cemre Candemir, Ali Saffet Gonul, Mustafa Alper Selver

**Affiliations:** 1 Department of Electrical and Electronics Engineering, Burdur Mehmet Akif Ersoy University, Burdur 15030, Turkey; 2 International Computer Institute, Ege University, Izmir 35100, Turkey; 3 Standardization of Computational Anatomy Techniques, SoCAT Lab, Ege University, Izmir 35100, Turkey; 4 Department of Psychiatry, Medical Faculty, Ege University, Izmir 35100, Turkey; 5 Department of Electrical and Electronics Engineering and Izmir Health Technologies Development and Accelerator (BioIzmir), Dokuz Eylul University, Izmir 35160, Turkey

**Keywords:** DWT, emotion, feature fusion, fMRI, LSTM, memory, multitask, ResNet, resting fMRI, task classification

## Abstract

One of the emerging fields in functional magnetic resonance imaging (fMRI) is the decoding of different stimulations. The underlying idea is to reveal the hidden representative signal patterns of various fMRI tasks for achieving high task-classification performance. Unfortunately, when multiple tasks are processed, performance remains limited due to several challenges, which are rarely addressed since the majority of the state-of-the-art studies cover a single neuronal activity task. Accordingly, the first contribution of this study is the collection and release of a rigorously acquired dataset, which contains cognitive, behavioral, and affective fMRI tasks together with resting state. After a comprehensive analysis of the pitfalls of existing systems on this new dataset, we propose an automatic multitask classification (MTC) strategy using a feature fusion module (FFM). FFM aims to create a unique signature for each task by combining deep features with time-frequency representations. We show that FFM creates a feature space that is superior for representing task characteristics compared to their individual use. Finally, for MTC, we test a diverse set of deep-models and analyze their complementarity. Our results reveal higher classification accuracy compared to benchmarks. Both the dataset and the code are accessible to researchers for further developments.

## 1. Introduction

Functional magnetic resonance imaging (fMRI) is one of the powerful noninvasive neuroimaging tools providing high spatial resolution to measure brain activity. Thanks to the functional imaging properties, it is possible to measure brain activity and explore the activated brain regions through the analysis of Blood Oxygenation Level Dependent (BOLD) signals. In addition to this, it is widely used in research studies to answer a wide variety of questions which are mainstays of clinical problems. These questions can be related to the effects of a given drug, the alteration of brain phycology due to a psychiatric disorder, or basic skills such as memory, speech, emotion, fear, and vision. Whether stimulated by cognitive, behavioral, or affective tasks with different characteristics, fMRI has the ability to show the associated and connected areas with the stimulus. It is also applicable when the subject lies still during rest.

Theoretically, obtaining BOLD signals from the functional image is calculated regardless of the type of the stimulus, i.e., the convolution of the hemodynamic response function with the given stimulus [1]. Conventionally, statistically relevant and correlated voxels above a specific threshold value are determined over time during the analysis of the functional data. Thus, these voxels are aimed to be mapped to the given stimuli, and the neuronal activity is observed [2]. In this approach, knowing the stimulus set is the fundamental condition.

The reversed question is, “is it possible to be able to infer the stimulus type by considering the neuronal activation patterns?” A challenging question appears since the signal patterns of the intrinsic or spontaneous neuronal activity are highly complex and cannot be identified visually or manually. However, through rapid developments in machine learning methodologies, it has been demonstrated that a reverse approach called “brain decoding” or “neuronal decoding” might answer this question. The underlying idea is to reveal the hidden brain patterns that correspond to the different structures of the fMRI tasks. Various studies showed that by using the brain activity signals, it is possible to identify some patterns such as speaking [3], auditory stimuli [4], motor imagery [5], visual images from simple objects to faces [6,7,8], imaged natural images [9], and intentions [10]. Reconstruction of the colored face images has also been reported with moderate accuracy [11]. Although some state-of-the-art studies offer using machine learning for behavioral coding [12], facial expression [13], or emotion, sentiment, and intensity prediction [14] on multitask frameworks, they are differentiated for not using any functional neuronal data.

On the other hand, most of the neuronal studies have been conducted for decoding only one of the neuronal activities, and classification of various tasks with distinctive cognitive states with multiple subjects is rarely reported [15,16,17]. Thus, multi-variate decoding is still an emerging field for studying brain functions [18,19].

**Figure 1:**
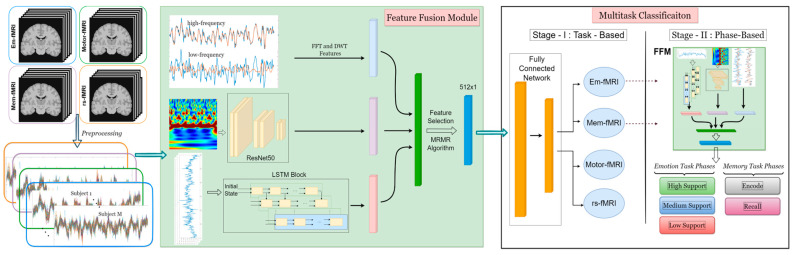
The proposed framework for automatic multitask classification of functional data. Raw functional images are acquired from cognitive (based on a memory task (mem-fMRI)), behavioral (based on a motor task (motor-fMRI)), and affective (based on an emotion task (em-fMRI)) fMRI tasks and a resting-state fMRI scan. All BOLD signals for N subjects (differs according to the dataset) are processed with the standard preprocessing steps with SPM. In FFM, the feature generation procedure is as follows: (i) each signal is decomposed into its low-frequency and high-frequency components with DWT and FFT; (ii) BOLD signals are converted into an image and fed into a ResNet-50; (iii) an LSTM block is employed to the BOLD signals. Once the three-step procedure is completed, all features are combined, and the most relevant features among them are selected with the MRMR algorithm. Finally, the obtained feature vector is fed into the Fully Connected Network (FCN) to conduct the task-based multitask classification (Stage I). For the sub-phase classification (Stage II), the FFM should be run again before the FCN.

In this study, we address the challenges and pitfalls associated with the Multi-Task Classification (MTC) problem. Furthermore, we extend our dataset and analysis to include the sub-phases of a specific task, which to the best of our knowledge have not been studied before. In this respect, we introduce a new dataset for MTC and propose a novel two-stage classification system for resolving associated challenges. The main contributions of our study are as follows:

(1) First of all, we introduce a new benchmark for MTC. It presents a new collection of fMRI datasets that bring together resting state, behavioral, cognitive, and affective functional tasks of healthy adults. The importance of introducing such datasets is due to the subjectivity of fMRI experiences (please see Section 2.1 for further details).

(2) We aim to provide foreknowledge about the activation patterns of signals on each different simulated task. To enable this, we also present new and original affective and cognitive tasks as part of the dataset discussed above (please see Section 2.2 for dataset details).

(3) We present a reliable, two-stage classifier that identifies the relationship between stimuli and BOLD signal. The first stage aims to make an accurate prediction of the type of stimulus from the activation patterns of BOLD signals. The second step is to determine to which sub-phase of the fMRI task a given piece belongs. Figure 1 illustrates the flowchart of the proposed algorithm.

(4) We propose a Feature Fusion Module (FFM) to extract and combine effective and unique features of the neuronal signals.

(5) We generate a signature for each task by combining hand-crafted features (frequency and time-frequency representations) with deep features and apply comprehensive tests to measure MTC performance. Moreover, we analyze the diversity and complementarity of different models to check the possible advantages of using ensembles.

The organization of the rest of the paper is as follows: First, we introduce proposed fMRI tasks and the dataset in Section 2. Then, we present the developed MTC framework and FFM in Section 3. After that, we illustrate and discuss the computational results and corresponding analysis in Section 4. Finally, we draw conclusions in Section 5.

## 2. fMRI Datasets and Properties

### 2.1. fMRI Acquisitions and Dataset Properties

As stated above, we present a new collection of fMRI datasets. Even though some fMRI datasets are shared as publicly available, they may not be suitable for use in a technical or hypothetical manner. The dataset consisting of a certain conducted type of task may not also be able to answer the questions in hypothesis. For example, emotions evoked visually and auditorily result in different activations in different brain regions. Thus, the acquisition of the data has crucial importance, in addition to the design and processing of the task and the analysis of the data. 

On the other hand, one of the most important restrictions of fMRI data is its productivity which stresses the importance of the public availability of the functional datasets. This limitation may be caused either by the subjects and/or by the scanners. The same results may not be acquired again even if the same fMRI task is used for different subject groups. The fMRI tasks, especially the affective ones, tend to be subjective since it aims to activate the memory/emotion-related areas of the brain. Accordingly, in such tasks, one of the expected results is that diversity is shown among the different participants (i.e., inter-subject variability). Aside from inter-subject variability, in the case of using the same scanner, the results may be impacted even due to a difference in technicians and their initial settings (intra-scanner reliability). This variability becomes even greater when we run the same task on different MRI scanners (inter-scanner reliability). A comprehensive study, comparing 1.5T and 3T, reports that although it does not guarantee uniform functional imaging results, 3T provides better quality and more advantages than 1.5T since it has better pulse sequences [20]. From a different perspective, another important issue is inter-scanner reliability. Several studies on rs-fMRI datasets point out that the reliability of the same scanner is higher than the inter-scanner reliability on test-retest scans in terms of several metrics [21,22].

In the presented dataset collection, we minimize both resolution and inter-scanner effects, as the images are taken with the same calibrations on a single scanner. At the same time, we eliminate the effects of human differences through images taken by a single MR technician. These issues state the difference and importance of the presented collection among the currently available datasets.

The datasets used in this study can be summarized as follows: Motor-fMRI is the behavioral task that reflects the neuronal activity while subjects perform a motor action (i.e., finger-tapping). Em-fMRI is the affective task, and it presents the emotional activity while subjects are stimulated with intentional emotional change phases. Mem-fMRI is the working visual memory task, i.e., the cognitive task, which consists of encoding, decoding, and resting blocks. rs-fMRI is the resting state fMRI dataset that presents spontaneous neuronal activity. We acquired the functional brain magnetic resonance images using 3 Tesla (3T) with Siemens Magnetom Verio Numaris/4, Syngo MR B17 whole-body scanner while subjects were performing the cognitive and affective tasks and during the resting state.

Additionally, a list of the datasets consisting of affective, cognitive, and behavioral tasks are summarized in Table 1. The datasets are selected from the most prominent and most possibly related ones. Here, they are presented by the task type, dataset name, subject number, scanner type, and dataset description.

Here, we emphasize that none of their combinations are the same for either stimuli type or scanner type as those we have presented. On the other hand, some of the public datasets may not provide a guarantee about the accuracy of their components such as event files [23]. As a matter of fact, such datasets could not have been analyzed correctly, thus causing restriction of the usability of existing available datasets.

**Table 1:** There are several publicly available datasets acquired from affective, cognitive, and behavioral tasks (or hybrid tasks, shown with an asterisk (*)). This table contains the prominent ones, along with most related datasets for various forms and special cases such as the ones that contain healthy and schizophrenic individuals.

Task	Dataset Name	#Subjects	Scanner Type	Description
Affective (Emotion)	Emotional regulation Task [24]	30	1.5T GE Signa Twin Speed Excite HD	Participant completes a task that induces emotional conflict while behavioral and/or physiological data is collected. Reduced negative emotional experience during cognitive reappraisal of aversive images.
	Affective videos [25]	11	3T Siemens Magnetom Trio	A task for determining whether affective states can be similarly identified when participants view dynamic naturalistic audiovisual stimuli.
	Emotional music comprehension/production in depression [26]	19	3T Siemens Skyra	Subjects listen to music passively or are asked to sing overtly to examine how neural processing of emotionally provocative auditory stimuli is altered in depression.
	EUPD cyberball [27]	20	3T Siemens Magnetom Verio	A task in which subjects view a set of balls interacting in a game. At some point, one of the balls is excluded from the game, simulating social exclusion.
Cognitive (Memory)	Incidental encoding task (Posner Cueing Paradigm) [28]	18	3T Signa MR scanner	A task in which the subject is creating new memories without purposely knowing that memorization is the task at hand. Their memories are created thorough working in their environment and picking up information in the process.
	Working memory in healthy and schizophrenic individuals [23]	40 (20 + 20)	3T Siemens Trio	A task in which participants view a continuous stream of letter stimuli. The object of the task is to identify letter repetitions that occur n-trials preceding the current stimulus. Letter n-back task.
	Visual imagery and false memory for pictures [29]	26	1.5T General Electric Signa HDe	A task in which subjects create mental images according to the given words and/or pictures of other common items.
*	Block tapping task [30]	30	NA	A task used for assessment of visual short-term memory and implicit visual-spatial learning. An examiner taps a series of blocks, and the subject must repeat it in the correct sequential order. If the sequence is correct, the examiner adds another tap to the next sequence. Voluntary and TMS-induced finger movements.
Behavioral(Motor)	Learning and memory: motor skill consolidation and intermanual transfer [31]	15	3T GE EXCITE 3 HD	Subjects tap their fingers according to a visual, auditory, or no cue.
	GDMotor [32]	29	NA	Goal-directed motor task.
	Visual and audiovisual speech perception [33]	60	3T Siemens Prisma	A behavioral lip-reading task. Visual and audiovisual processing of single words in adult participants. Words were presented in quiet for auditory only, visual only, and audiovisual stimuli.
	Simultaneous MRI-EEG during a motor imagery neurofeedback task [34]	30	3T Siemens Verio	A multimodal dataset of EEG and fMRI acquired simultaneously during a motor imagery NF task, supplemented with MRI structural data.

The parameters of the imaging procedures are flip angle (FA) = 90° and bandwidth = 2232 Hz/pixel for all scans. Repetition times (TR) and echo times (TE), the field of views (FOV), and slice numbers vary according to the tasks. Except for the motor task, all imaging procedures were conducted by SoCAT Research Lab in Ege University, Turkey. The participants were right-handed, healthy, university student volunteers. The motor-task dataset regarding the suitability of the task design, imaging procedures, etc., was acquired, after a meticulous selection, from the rt-me-fMRI project of the Eindhoven University of Technology and is publicly available [35].

### 2.2. Dataset Descriptions

#### 2.2.1. Resting State fMRI (rs-fMRI)

Unlike task-based fMRI models, rs-fMRI focuses on spontaneous and intrinsically generated neuronal activity in BOLD signals. During the scan, the participants are not stimulated by any task, and they are asked to be comfortable, lie still, and not to think about anything. The eyes of the participants may be open or closed. The regions that are active in the brain in a resting state are called the default mode network, and no activation is expected in areas outside of this network. In this dataset, the rs-fMRI task lasts 9 min in total. The total brain imaging data consist of 37 slices, 64 × 64 matrix, FOV = 192 mm × 192 mm, voxel size 3 × 3 × 3 mm, slice thickness 3 mm, TE = 30 ms, TR = 3000 ms. 180 image series are acquired for 23 subjects (f:11,m:12,μ=22.54±1.02).

#### 2.2.2. Emotion fMRI (em-fMRI)

The emotion-based affective em-fMRI dataset is obtained through a social support fMRI task [36]. The task consists of a game aimed at triggering the alteration of emotional states through different levels of social support. During the task, the participant and his three friends play a guessing game against a rival and win some money at the end. The whole game period consists of three main support stages. The first stage is the high-support stage, and participants win 80% of the game thus feeling the support of their friends. However, the last part is the low-support stage, and participants lose 80% thus not feeling any support from their friends. The middle stage is the medium-support stage that can be thought as a transition stage between first and last stages. For each voxel, 600 image series are acquired with 37 slices, 64×64 matrix, the voxel size 3 mm×3 mm×3 mm, 3.5 mm slice thickness (with 1 mm gap), 200 mm×200 mm FOV, TE = 30 ms, TR = 3000 ms. The task lasts about 30 min (1818 s), and the data of 14 participants are marked as valid (f:7,m:7,μ=21.72±1.6).

#### 2.2.3. Motor fMRI

This is a finger-tapping experiment to identify motor-related regions in the brain. The motor-fMRI task has a block design that includes repetitive rest and movement parts. In the rest period, subjects stand without any movement for 20 s, and thereafter subjects are asked to perform repetitive finger opening and closing movements for 20 s during the movement period. Each of the rest and movement periods are repeated 10 times. A series of 200 images of every voxel is acquired with 34 slices with a matrix of size 64 × 64, voxel size = 3.5 mm × 3.5 mm × 3.5 mm, TR = 2000 ms, TE = 14 ms, FOV = 224 mm × 224 mm; 3.5 mm slice thickness for 28 subjects $\left(f:8,m:20,\mu =24.9\pm 4.7\right).$

#### 2.2.4. Memory fMRI (mem-fMRI)

Mem-fMRI has a block design consisting of sequential resting, encoding, resting, and recall phases. During the encoding phase, subjects are asked to record the given name associated with the given faces. And during the recall phase, subjects are asked both to decide whether they are familiar with the faces and whether the name-face pairs are true or not. The total brain imaging data consists of 37 slices, 64 × 64 matrix, FOV = 192 mm × 192 mm, voxel size = 3 mm × 3 mm × 3 mm, slice thickness = 3 mm, TE = 30 ms, TR = 3000 ms for 20 subjects (f:11,m:9,μ=23.35±1.04).

### 2.3. Signal Preprocessing

The standard preprocessing steps of the functional data were performed with the Statistical Parametric Maps (http://www.fil.ion.ucl.ac.uk/spm/ accessed on 1 June 2022) (SPM) toolbox, which runs on the MATLAB platform. All functional images were corrected for involuntary head motion, which is known as the realignment step, and afterwards the slices were synchronized temporally in the slice timing step. Later, the structural scans of the subjects were registered to the mean images of fMRI scans, which is referred to as the co-registration. The next preprocessing step was the segmentation, where the brain is separated from its surrounding tissues. During segmentation, the structural image was also normalized to a global standard space, which is the standard Montreal Neurological Institute (MNI) single-subject template. Finally, normalized images were spatially smoothed with an 8 mm isotropic Gaussian kernel.

### 2.4. ROI Selection and Signal Extraction

**Figure 2:**
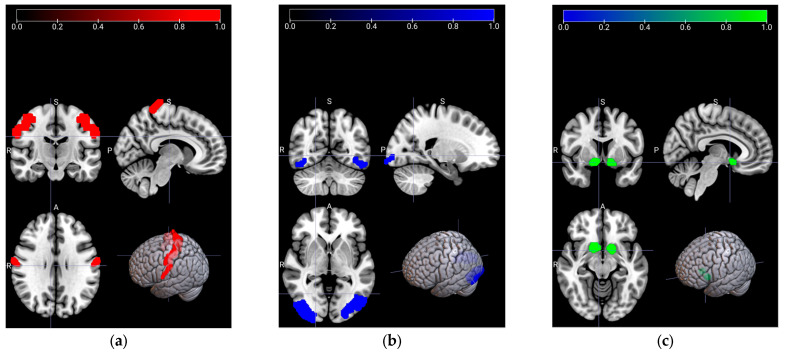
The region-of-interest (ROIs) of the task-related areas are illustrated in coronal, sagittal (top row), and axial (bottom row-left) anatomical sections, respectively, as well as with the locations in the rendered brain (bottom row-right). (**a**) Nucleus Accumbens (NAcc), shown in green, is associated with the reward-related activities; (**b**) Broadmann Area-4 (BA4), shown in red, is related to motor movements; (**c**) Occipital Face Area (OFA), shown in blue, is related to face processing and recognition.

Region-of-Interest (ROI) selection is a sophisticated process, so it should be evaluated very carefully by the experts according to the hypothesis of the research question. The first step in determining the ROIs is to complete the first-level (individual level) for each subject and each task. The hypotheses are searched by the contrast vectors, which are based on the statistical inferences and on the functional data. Since the fMRI tasks are built on different hypotheses, the contrast vectors also differ for each task. This results in distinct ROIs among the fMRI tasks. The main point for the ROI selection is to be able to conduct the second-level (group level) analysis after completing the first-level analysis. Thus, the activation maps exhibit the active areas for the entire group. The signals have been revealed by experts from the determined ROIs, which are illustrated in Figure 2. Nucleus Accumbens (NAcc), associated with reward-related behaviors, is given in Figure 2a. In Figure 2b, Broadmann Area 4 (BA4) is shown, which is related to the motor movements for Em-fMRI. Finally, Occipital Face Area (OFA), which plays an important role for face processing and recognition, distinguishing familiar faces, and responding to face stimuli, is presented for the mem-fMRI in Figure 2c. For the rs-fMRI, the signals have been gathered from all the determined ROIs additionally.

The selected ROIs have been specified as having a cluster size > 10 adjacent voxels with the threshold of *p* < 0.05 with Family Wise Error (FWE) corrected on SPM12. ROI masks have been generated using the WFU Pick Atlas. As the last step, all acquired neuronal signals that constituted the signal pool have been normalized to zero mean and detrended before being fed into the proposed model. In total, 19,221 signals are included in the data pool.

## 3. Proposed Multitask Classification Method and Feature Fusion Module

### 3.1. Feature Fusion Module (FFM)

A typical functional brain scan contains n x m x k number of voxels, and this number is generally more than 100,000. When each and every voxel is considered, the resulting time series signal is too large to process one by one. Moreover, it becomes error-prone because of the possible conflicting sights of the experts. Furthermore, the data size increases considerably in multi-subject and multitask scans. On the other hand, due to the nature of the functional data, signals contain high amounts of noise and sporadic artifacts. For this reason, the signal-of-interest may be suppressed, and valuable information could be missed during the analysis. Thus, reducing the feature size is a fundamental processing step before applying a machine learning methodology and the common point of view from various neuroimaging studies [37,38]. In this way, the overfitting problem is prevented, and the classification accuracy can be increased.

Considering the complexity of multitasking data, selecting the representative features becomes an essential component of the proposed method. Therefore, to cope with the entire signal set, we present a feature fusion module (FFM) as a combined feature extraction method. FFM assumes that all the information of the neural patterns is involved in the signals, and it aims to concatenate a feature set that reflects the unique characteristics of a given task. As shown in Figure 1, FFM is constructed from four methods: Fast Fourier Transform (FFT), Discrete Wavelet Transform (DWT), Residual Neural Network (ResNet), and Long-Short Term Memory (LSTM). Thus, it is ensured that the most relevant features can be represented with FFM since the BOLD signals have complex and diverse structures. The FFM steps are given as follows:

1. Fast Fourier Transform: Fourier Transform (FT) is one of the main techniques for extracting frequency components in a signal by projecting the signal onto the basis functions. On the other hand, FFT is an algorithm used to compute discrete Fourier transform in an efficient manner in terms of computational complexity. FFT is employed in order to extract the frequency components contained in the BOLD signals by representing them in the frequency domain.
2. Discrete Wavelet Transform (DWT): It is well known that DWT can successfully analyze complex problems, as the analyzed signal provides both frequency and position information by using multi-resolution analysis [39]. It provides a coarse-to-fine strategy so that it is very useful for characterizing different structured data. In FFM, DWT is used to decompose the BOLD signals into a low-frequency signal and a high-frequency signal (i.e., multiband signals).
3. ResNet: ResNet-50 is a residual network containing 50 layers. Residual connections in the network prevent the model from exploding and vanishing gradient problems. It is applied for image classification tasks and trained by using more than a million images with 1000 classes from the ImageNet [40] database. The input size of the ResNet-50 network for images is 224×224.
4. LSTM: LSTM, proposed in [41], is a deep learning architecture widely used for time series applications. It is proposed in order to overcome the vanishing gradient problem of Recurrent Neural Networks. An LSTM memory has three gates which are responsible for controlling the information flow throughout the memory. These gates are named input, output, and forget gates. Input and output gates control the flow of information, and the forget gate resets the memory of the LSTM cell when the cell memory is not used anymore. The input gate also controls the cell state together with the forget gate. Assume that $x_{i}$ is the input signal at time t; let the input gate, the output gate, and the forget gates be denoted as $i_{t},o_{t}$, and $f_{t}$, respectively. Then, the input gate can be expressed as
  (1) $$i_{t}=\sigma \left(W_{i}\left[h_{t-1},x_{t}\right]+b_{i}\right)$$
  Moreover, the output gate is given by
  (2) $$o_{t}=\sigma \left(W_{0}\left[h_{t-1},x_{t}\right]+b_{0}\right)$$
  and the forget gate is written as
  (3) $$f_{t}=\sigma \left(W_{f}\left[h_{t-1},x_{t}\right]+b_{f}\right)$$
  Here, the parameters given by $W_{i},W_{0},$ and $W_{f}$ notations are input, output, and forget weight parameters. On the other hand, $b_{i},b_{0},$ and $b_{f}$ utilized above the equations to express cell gates represent the bias parameters. $h_{t}$ and $c_{t}$ are the hidden and cell states, and they are expressed as the following forms, respectively:
  (4) $$c_{t}=i_{t}⊙\tilde{c_{t}}+f_{t}⊙c_{t-1}$$
  (5) $$h_{t}=o_{t}⊙\tanh\left(c_{t}\right)$$
5. MRMR: The MRMR algorithm is one of the feature selection algorithms based on the filter method. Filter method-based feature selection algorithms are computationally efficient methods, and they can be generalized to different machine learning models [42]. The MRMR algorithm was proposed in [43] to find an optimal feature subset by maximizing the relevant and minimizing the redundancy of feature set.

Let $f_{k}^{n}$ denote a single feature obtained from a single sample where k=1,2,…,K represents the feature number and n=1,2,…,N indicates the sample number. Thus, the vector of $F_{k}=[f_{k}^{1},f_{k}^{2},f_{k}^{3},\ldots ,f_{k}^{N}]$ shows the *k-*th feature collected from all samples. Therefore, ${\left\{F_{k}\right\}}_{k=1}^{K}$ denote all features of the samples in a dataset. Let S represent the selected feature subset, then redundancy is defined as [43,44]

(6) $$\min\;W,\;W=\frac{1}{{\left|S\right|}^{2}}\sum_{F_{i},F_{j}\in S}I\left(F_{i},F_{j}\right)$$

where $I\left(.\right)$ represents the mutual information, $F_{i}$ is a feature from subset S, i.e., $F_{i}\in S$, and $F_{j}$ is a feature currently not selected, i.e., $F_{i}\notin S$. $In\left(6\right),$ $\left|S\right|$ represents the number of selected features in S. On the other hand, relevance, as follows [42,43,44], is given by

(7) $$\max\;V,\;V=\frac{1}{\left|S\right|}\sum_{F_{j\in S}}I\left(Y,F_{j}\right)$$

Here, Y is the target classes given by $Y=\left[y_{1},y_{2},\ldots ,y_{K}\right]$.

The MRMR algorithm optimizes the criteria given in (6) and (7) simultaneously, and it is accomplished by combining them in a single criterion. The two simplest combination criteria can be formulated as [43]

(8) $$max\left(V-W\right)$$

(9) $$max\left(V/W\right)$$

Finding the solution of the above criteria requires $O\left(N^{\left|S\right|}\right)$ as researched by [43]. Instead, the algorithm runs in a more efficient way. The first feature is selected by considering the largest relevance, i.e., the formulation given in (7), and is added to the selected feature set, S. Then, other features are selected in incremental progress. Let Ω denote the set of all features of the samples. Thus, the feature set, except for the already selected features, can be given as

(10) $$\Omega_{S}=\Omega -S$$

Incremental progress runs by optimizing the following conditions [43]

(11) $$\underset{F_{j}\in \Omega_{S}}{\max}I\left(Y,F_{j}\right)$$

(12) $$\underset{F_{j}\in \Omega_{S}}{\min}\frac{1}{\left|S\right|}\sum_{F_{i}\in S}I\left(F_{i},F_{j}\right)$$

Above, the condition given in (11) is equivalent to the condition in (7). On the other hand, the condition in (12) is an approximation of the redundancy condition in (6) [43]. Finally, in order to select the new feature, the combination of redundancy and relevance given in (8) and (9) become [44].

(13) $$\underset{F_{j}\in \Omega_{S}}{\max}\left[I\left(Y,F_{j}\right)-\frac{1}{\left|S\right|}\sum_{F_{i}\in S}I\left(F_{i},F_{j}\right)\right]$$

(14) $$\underset{F_{j}\in \Omega_{S}}{\max}\left[I\left(Y,F_{j}\right)/\frac{1}{\left|S\right|}\sum_{F_{i}\in S}I\left(F_{i},F_{j}\right)\right]$$

Here, (13) and (14) are named Mutual Information Difference (MID) and Mutual Information Quotient (MIQ) criteria, respectively.

### 3.2. Multitask Classification Model

In this study, we are concerned with the classification of fMRI signals by employing deep learning-based methods. In addition, we utilize feature fusion and feature selection methods in order to both increase the classification performance of the model and to reduce the dimension of the data. The classification of signals is used for the categorization of a related signal into sub-categories by using some inherent features of the dataset. Assume a sequence $X=[x_{1},\ldots ,x_{T}]$ represents s signal with $x_{t}\in R^{d}$,where *d* is the number of dimension of data, $x_{t}$, at time *t*. Thus, finding a nonlinear mapping function, f(.), matching a sequence with a predefined labeled class is the main concern of a given classification problem. Here, we propose employing cascaded and ensemble models for the classification of fMRI signals.

The flowchart of the fMRI signal classification system proposed in this paper is given in Figure 1. It consists of two main classification stages. The first stage, named Stage I, is the task classification stage, and we determine the Em-fMRI, rs-fMRI, motor fMRI, and Mem-fMRI classes utilizing the acquired fMRI signals. Additionally, in Stage II of the proposed system, we classify Em-fMRI and Mem-fMRI signals into the sub-tasks the participant performs during the experiments. An acquired Em-fMRI signal includes three different emotions of the participant. Therefore, the Em-fMRI signal is classified into high-support, medium-support, and low-support classes that reflect participants’ emotional changes during the experiment. We also classify a Mem-fMRI signal into two different groups, encoding and recall classes, which indicates the main phases of a Mem-fMRI task. 

A BOLD signal acquired from a participant does not allow us to classify the signal into sub-tasks or phases using only one stage system. This is the reason why we employ a two-stage classification system. As stated above, an Em-fMRI signal includes three phases: high-support, medium-support, and low-support. Therefore, before determining the phases of an acquired signal, we need to know whether the signal is classified as the Em-fMRI task in Stage I. Three different phases are sequentially and equally spaced in the EM signals. Thus, the phase classification of the Em-fMRI signal is impossible without a two-stage system because of the structure of data acquired from participants during the experiments.

Our proposed deep learning-based system (Figure 1) is used in Stages I and II. The system is built up in order to utilize the feature extracted from 1D and 2D spaces. In order to extract 1D time-dependent features, time series fMRI signals are presented to the LSTM model. At the same time, FFT and eight-level DWT coefficients of the time series signal are calculated to utilize the frequency information of the signal. The third model used in the proposed system is the ResNet-50 network. The 2D image input of ResNet50 are scalogram images that contain time-frequency information about BOLD signals and are obtained by employing a continuous wavelet transform (CWT). The frequency of BOLD signals acquired by participants during the experiment may change over time based on the given task. Especially the frequency variation can be observed during the transition from one phase to another in the experiment. Therefore, we use features obtained from scalogram images to grasp information about different sub-tasks. Finally, features obtained from time-series signals and scalogram images using LSTM and ResNet-50 network, respectively, are concatenated with FFT- and DWT-based features.

As stated in Section 3.1, $f_{k}^{n}$ indicates a single feature obtained from a single sample. Then, $f^{n}=[f_{1}^{n},f_{2}^{n},f_{3}^{n},\ldots ,f_{K}^{n}]$ denotes all the features obtained from a single sample. The features of a single sample extracted using the two-level cascaded LSTM network, the ResNet-50 model, and FFT- and DWT-based methods shown in Figure 1 can be given by $f_{L}\in R^{100\times 1}$, $f_{R}\in R^{2048\times 1}$, and $f_{F}\in R^{607\times 1}$. Here, sub-indices L, R, and F represent LSTM, ResNet-50, and FFT-DWT-based features, respectively. In FFM, all features of a single sample are concatenated and represented by $f_{FFM}\in R^{2755\times 1}$. 

In our proposed system, we also employ the feature selection method in order to both increase the performance of the system and reduce irrelevant data which increases the computation time. Therefore, after accomplishing feature fusion, we employ a minimum redundancy maximum relevance (MRMR) method on concatenated features extracted using different networks. Using this method, the number of features is reduced to 512 and can be represented by $f_{MRMR}\in R^{512\times 1}$. Finally, selected features are presented to a two-layer, fully connected network to classify the BOLD signals.

**Figure 3:**
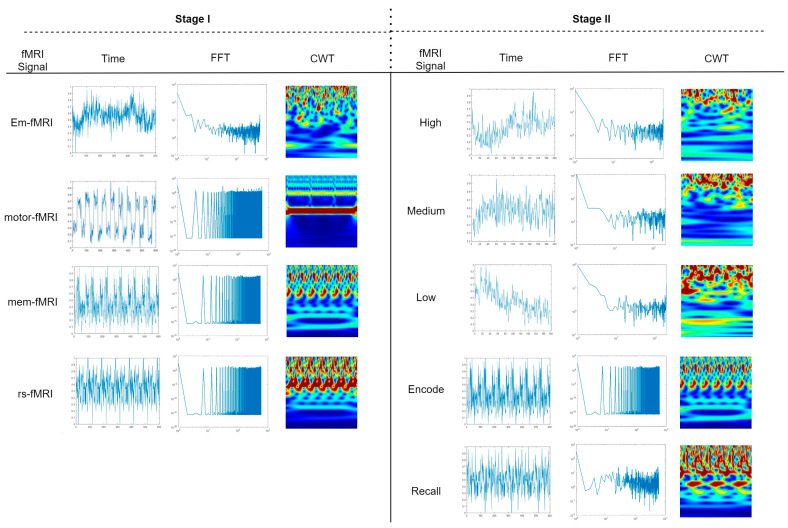
BOLD signal of a selected voxel generated during fMRI acquisition and its FFT (logarithmic scale) sequences and CWT images. Stage I and Stage II represent the stages in which the signals are used in our classification systems.

Figure 3 shows the 1D BOLD signal acquired from participants, the FFT of the signals, and the scalogram images obtained by employing continuous wavelet transform (CWT) for different tasks and phases used in Stage I and Stage II.

## 4. Analysis and Results

All simulations we employ in this paper are performed on a PC having i7-9750H CPU with a processor speed of 2.6 GHz and a memory of 16 GB. In order to use the computation power of GPU, algorithms are run by utilizing the NVIDIA GTX 1660 Ti GPU with a memory of 6 GB.

In our experiments, since the length of fMRI signals acquired by participants are different, they are padded before being fed into the deep neural network at the beginning of Stage I. This way, all fMRI signals presented to the network in Stage I become 1×600 vectors, and therefore, each signal can be represented by $X=[x_{1},\ldots ,x_{T}]$ where $x_{t}\in R,t=1,\ldots T$ and T=600.

The dataset in this paper can be given by $D=\left\{\left(X_{n},Y_{n}\right)|X_{n}\in R^{1\times T},Y_{n}\in \{1,C\}\right\}\;$ where n=1, …, N and $X_{n}$ represent the individual sample in the dataset. Here, N, T, and C are total number of samples, the size of each sample, and the number of classes, respectively. The total number of samples, that is, the number of collected signals from the participants, N, is 19,221, and the size of each sample, T, is 600. Training and test samples are divided using a k-fold cross validation approach by assigning the parameter k=12. Therefore, 17,620 signals are used for training, and 1601 signals are used for testing in each experiment, and the 12 experiments are performed for performance measurements. The number of classes, C, is four at Stage I and five at Stage II.

In the experiments performed in Stage I, we employed a two-layer LSTM network to extract features from one-dimensional fMRI signals. In order to find the optimal model parameters for a two-layer LSTM network, we search the hidden units in {50,75,100,125,150,175,200,250}, learning rate, lr, in $\left\{10^{-2},5\times 10^{-3},10^{-3},5\times 10^{-4},10^{-4}\right\}$, and the number of epochs in {200,300,…,1000} on the validation set. At the same time, scalogram images of the corresponding fMRI signals are given to the pretrained ResNet-50 network. We extract scalogram features from the ResNet-50 model by using the output of the max-pooling layer before the final FC layer. Thirdly, FFT and eight-level DWT coefficients of fMRI signals are calculated. Finally, we concatenate the time-dependent features obtained from the LSTM layer, image-based features obtained from ResNet-50, and the FFT-DWT-based features. To be more similar to real-life scenarios, we use fMRI signals acquired from one participant at each training process as test data. On the other hand, fMRI signals of the other participants are considered as training data. 

Numerical results for the task classification experiment are given in Table 2. Classification performances of LSTM, Resnet-50, and feature fusion and feature selection based on proposed models are computed in terms of precision, recall, and *F_1_* score metrics.

In Stage II, we aim to determine the emotions and memory activities of participants using their Em-fMRI and Mem-fMRI signals, respectively. For this, signals determined as Em-fMRI and Mem-fMRI in Stage I are classified into their sub-tasks using the given deep learning-based methods. Emotions of a participant are classified into high-support, medium-support, and low-support. Assume that X is a 1×600 vector and that it represents the Em-fMRI signal acquired from a voxel for a particular participant. Thus, all three emotions of a participant are included in the Em-fMRI signal, X. Moreover, the experiment is designed in such a way that the emotional changes have the same time intervals. Therefore, we divide the signals x into equal time intervals, $x_{1}$, $x_{2}$, and $x_{3}$, before feeding the network. Hence, $x_{1}$, $x_{2}$, and $x_{3}$ become 1×200 vectors obtained from the Em-fMRI signal. In order to classify the signal X into sub-phases, we employ a similar FFM network except for the parameters. The LSTM model in the FFM network has two layers with 150 and 100 hidden units and dropout layers with 0.2 and 0.1 dropout ratios, respectively. Batch size and epoch numbers are chosen as 256 and 500, respectively. Table 3 shows the classification performances of LSTM, ResNet-50, and the proposed system in terms of precision, recall, and F1 score metrics.

Table 4 shows the overall accuracy of the task classification and sub-task classifications accomplished in Stage I and Stage II, respectively. We can see from Table 4 that the proposed model outperforms the LSTM and ResNet-50 models in cases where they are individually used for classification purposes.

To compare the performance of the FFM model, the diversity measures of all models are analyzed and presented in Table 5. Considering Table 5, it can be seen that the FFM models increase the classification performance by targeting the test data which other models miss. For example, in Stage I, the proposed method and LSTM both classify the 18,375 samples correctly (hit), whereas both methods classify 271 samples incorrectly (miss). However, the superiority of the proposed method can be seen where LSTM misses 504 samples, while FFM only misses 71. Similar observations can be made for ResNet-50 in Stage I. On the other hand, the FFM model especially correctly classifies the signals of participants to determine the emotions and memory activities of participants in Stage II, while other models cannot. In Stage II, LSTM misses 606 and 307 samples in emotion and memory sub-phase classifications, respectively, whereas the FFM-based model has 73 misses in the emotion task and zero misses in the memory task.

In Stage I, although the LSTM model performs better than the other models for motor fMRI signals, the proposed system outperforms the other methods in the fMRI task classification stage. The notable difference of our model can be seen in the emotion and memory activity classification in Stage II. The overall accuracies of the systems also show the advantage of our method. Additionally, we present the diversity of models to confirm the validity of our model. Diversity results indicate that the FFM-based model can correctly classify some of the signals, while other methods miss them. We believe that this result is also an indicator of the power of FFM and the feature selection algorithm.

## 5. Conclusions

It is well known that brain signals contain specific information about neuronal activity patterns. However, since the nature of BOLD signals is complexly structured, it is difficult to reveal these hidden patterns from the acquired signals. Even though their identification is possible thanks to the emergence of machine learning methods, multitask classification is still a challenging problem due to various pitfalls, which are not analyzed in detail in the literature since most of the state-of-the-art studies report single neuronal activity tasks.

In this paper, we propose a novel two-stage automatic multitask classifier for functional neuroimaging data containing various structured fMRI tasks. In the first stage, the system detects the main fMRI task (such as emotion, memory, motor, and resting) from a randomly given BOLD signal. Then, at the second stage, the system aims to categorize a sub-phase of the main task (such as high, medium, or low social support sub-phases for the emotion task, and the encoding or decoding phase for the memory task). 

To accomplish this, we propose a Feature Fusion Module (FFM) that creates a unique signature for each task by combining hand-crafted features with deep ones. We show through extensive analysis that FFM is able to reveal the characteristics of sub-phase signals very effectively. To the best of our knowledge, this is the first automatic multitasking classification method including both phase and sub-phase identification.

Evaluation results show that FFM with the feature selection method significantly increases the classification performance. The performances of LSTM, ResNet-50, and proposed FFM-based models are evaluated with precision, recall, and f-1-score metrics. For Stage I, it can be seen that the proposed FFM-based model can distinguish the given tasks, with over ~96% success compared to the other methods. The performance difference between the proposed and other methods becomes even more apparent in the Stage II classification. It is shown that the proposed method outperforms in emotion and memory phase classification tasks with at least 92.55% performance precision. On the other hand, the overall classification performance of the FFM-based model reaches 98.26% for task, and 96.02% and 100.0% for sub-phase classifications for emotion and memory, respectively. According to the diversity measures of the models, the proposed model has the advantage of boosting classification performance by targeting test data that other models miss. It also points out that it provides a high potential for distinguishing more complex tasks among the outnumbered subject groups.

Diagnosis, follow-up, and personalized treatments are still big issues in many psychiatric and some neurological diseases. There is an attempt to overcome current problems through clinician observation and scales based on patients’ self-reports. Therefore, information from any other tool like fMRI is important if the information is properly classified and interpreted. The fMRI signals obtained from the various tasks are also consistent with the extrinsic mode network (EMN), which is one of the brain networks activated with various types of stimuli (memory, attention, conflict resolution, etc.) and negatively correlated with the resting state [45]. In this context, it could be also possible to distinguish signals whether they belong to the EMN or the default mode network (DMN).

Here, it is also worth discussing the methodology and the study from several points of view. Although machine learning techniques yield very successful results for segmentation and classification problems, it should be remembered that these techniques are data-specific due to being data-dependent. Therefore, a method developed for one dataset cannot be guaranteed to work for another one. In the best case, it requires fine-tuning. In this study, all volunteers participating in the study were healthy, and a diagnostic-specific classification was not the aim in this context. The proposed FFM method was carefully designed to work on fMRI signals, and it can be said that it is technically possible to adapt it to the signals acquired from diagnostic-specific fMRI tasks as well. However, nothing definite can be said about its performance without further tests.

The other topic is the applicability in clinical translation. In medical practice, it is known that self-evaluation tests are frequently used methods for their benefits in diagnosing psychopathology. The common practice in clinical fMRI research is to evaluate the scales with the neural correlates obtained with an MRI scan. Undoubtedly, these tests aimed to be applied for the accuracy of the clinical diagnosis should also be selected appropriately by specialists. fMRI tasks should also be designed as an experiment to reflect this. On the other hand, the combination of clinical self-assessment scales and simultaneous fMRI acquisition studies are also interesting and attractive, especially in specifying the diagnosis [46]. When such methods are combined with novel methods, they can be guiding, especially in clinical applications. In addition to this, the proposed FFM-based method can also contribute to such specific diagnostic studies. It is technically possible to use the method to determine the disease relationship in the outputs obtained when disease-related scales, such as Beck Depression Inventory, are used together with fMRI. However, for accurate results, careful analysis is required. New and current assumptions about brain–behavior relationships, such as whole-brain or complexity, must also be addressed [47]. To improve further studies, the largest possible number of variables that will affect brain activation must be taken into account. Self-evaluation tests can also be included in these variables in this manner. Eventually, with technical advances, fMRI results and clinical results will be complementary methods.

## Figures and Tables

**Table 2:** Classification performances of models for behavioral, cognitive, and affective tasks classification experiment.

Model	Metrics	fMRI Task (%)			
		Emotion	Memory	Motor	Resting
LSTM	Precision	**99.13**	94.89	**100.00**	96.18
	Recall	97.22	91.17	95.83	98.04
	f1-Score	**98.11**	92.48	97.22	97.02
ResNet-50	Precision	96.54	97.39	**100.00**	96.69
	Recall	99.02	90.80	98.75	98.91
	f1-Score	97.67	93.79	**99.32**	97.76
Proposed	Precision	96.62	**99.34**	95.87	**98.29**
	Recall	**99.84**	**94.21**	**99.58**	**99.65**
	f1-Score	98.07	**96.54**	97.31	**98.95**

**Table 3:** Classification performances of models for emotion and memory phase classification experiments.

Model	Metrics	Emotion (%)			Memory (%)	
		High	Medium	Low	Encode	Recall
LSTM	Precision	78.33	76.11	83.63	97.30	97.47
	Recall	81.70	70.92	83.17	94.18	98.33
	f1-Score	78.73	72.60	81.78	94.68	97.74
ResNet-50	Precision	91.15	84.85	88.51	**100.00**	**100.00**
	Recall	88.32	82.60	91.67	**100.00**	**100.00**
	f1-Score	89.17	83.27	89.96	**100.00**	**100.00**
Proposed	Precision	**94.93**	**92.57**	**92.55**	**100.00**	**100.00**
	Recall	**95.02**	**87.99**	**96.32**	**100.00**	**100.00**
	f1-Score	**94.79**	**89.94**	**94.32**	**100.00**	**100.00**

**Table 4:** Overall accuracy of models for task classification in Stage I, and emotion and memory phase classification experiments in Stage II.

Model	Task and Sub-Task Classification (%)		
	Stage I	Stage II-Emotion	Stage II-Memory
LSTM	96.02	78.59	96.21
ResNet-50	96.85	87.53	**100.00**
Proposed	**98.26**	**96.02**	**100.00**

**Table 5:** Diversity of classifiers models.

	Task		LSTM		ResNet-50	
			Hit	Miss	Hit	Miss
Proposed	Stage I	Hit	18,375	504	18,482	397
		Miss	71	271	118	224
	Stage II-Emotion	Hit	2813	606	3102	317
		Miss	73	180	112	141
	Stage II-Memory	Hit	4604	317	4921	0
		Miss	0	0	0	0

## Data Availability

Available upon request.
